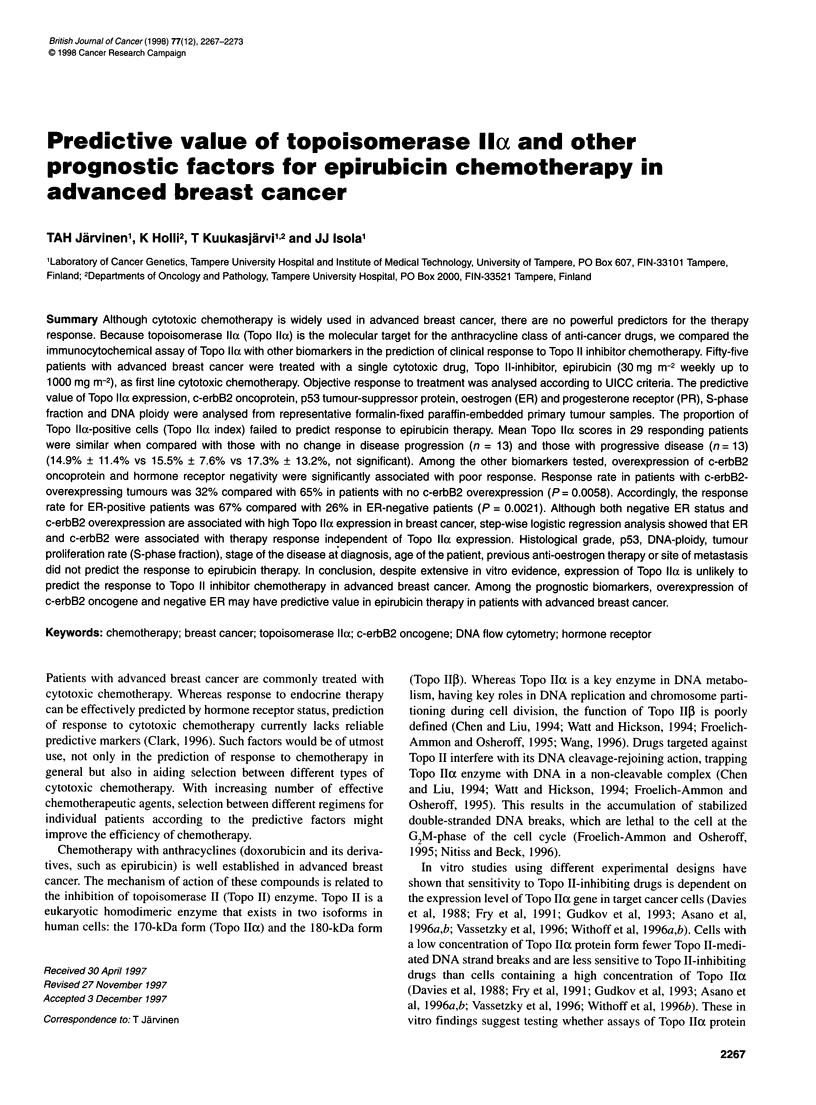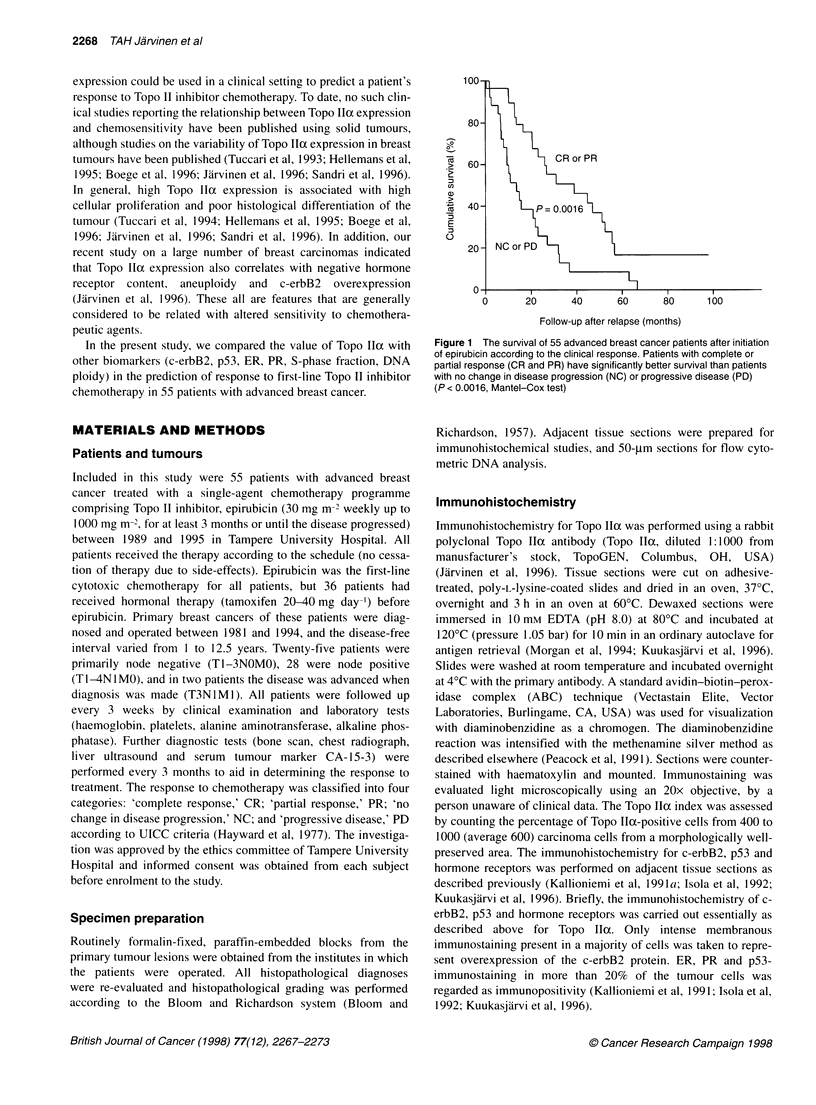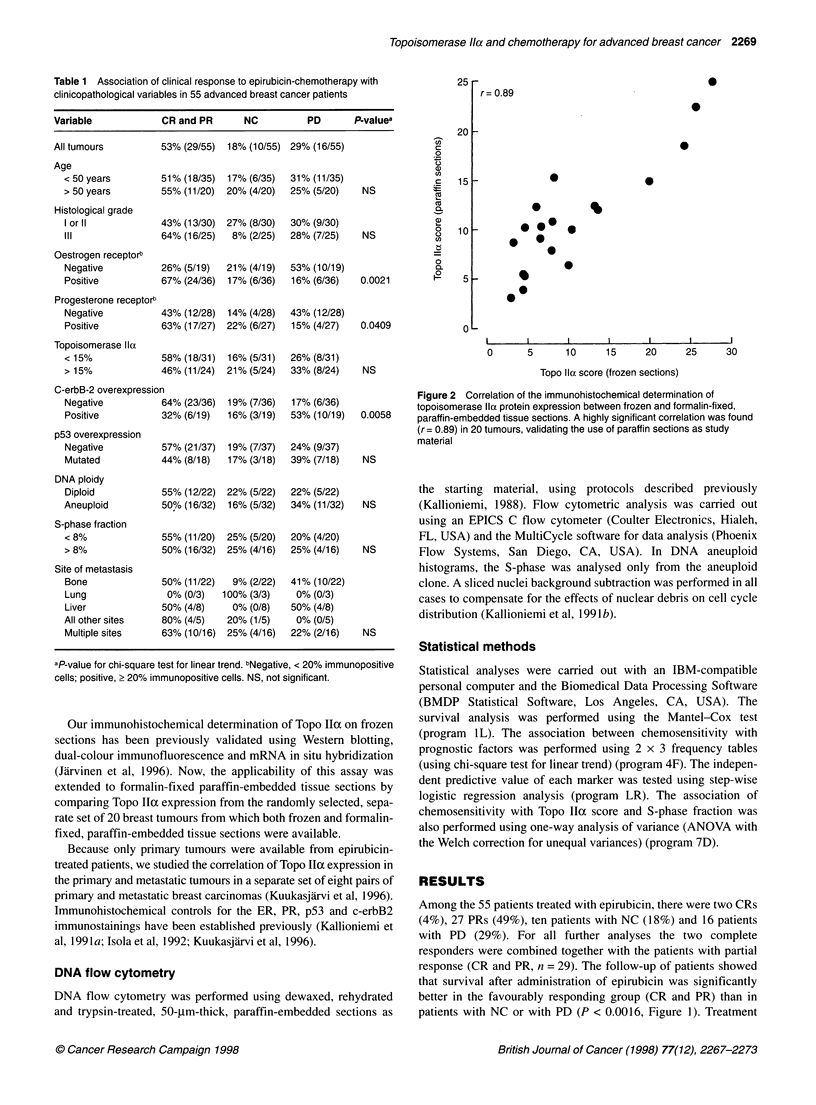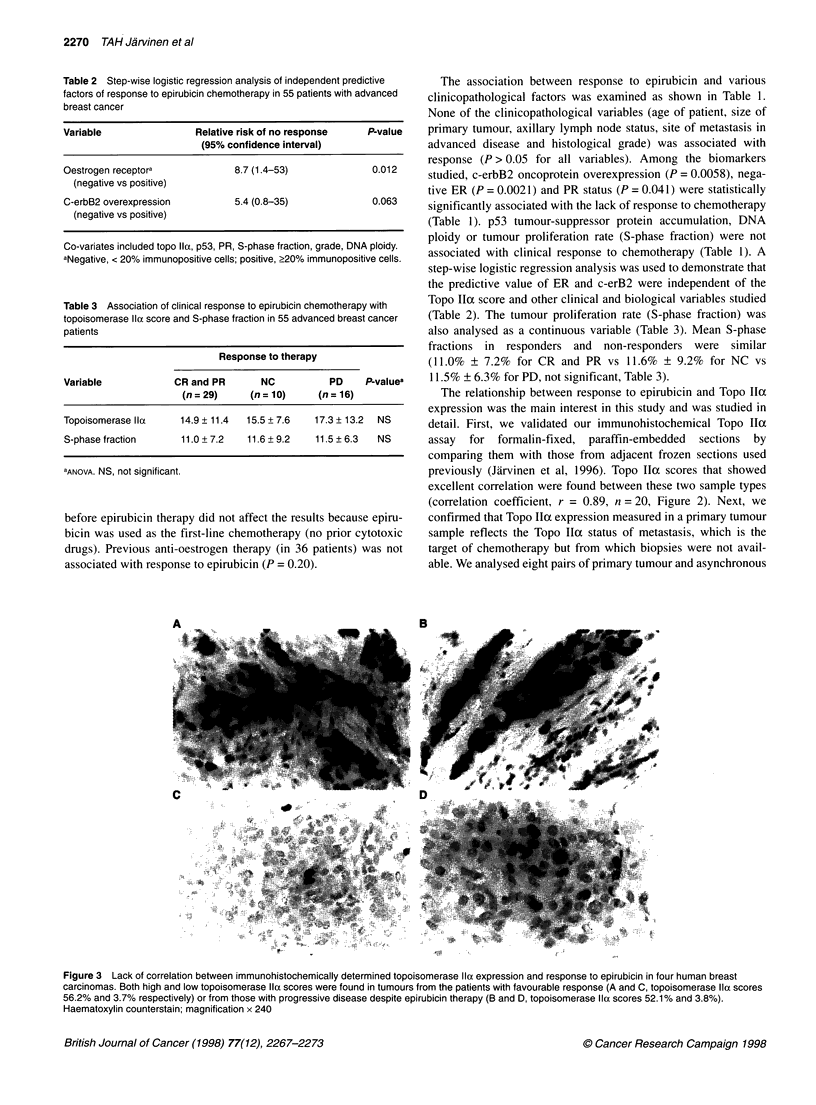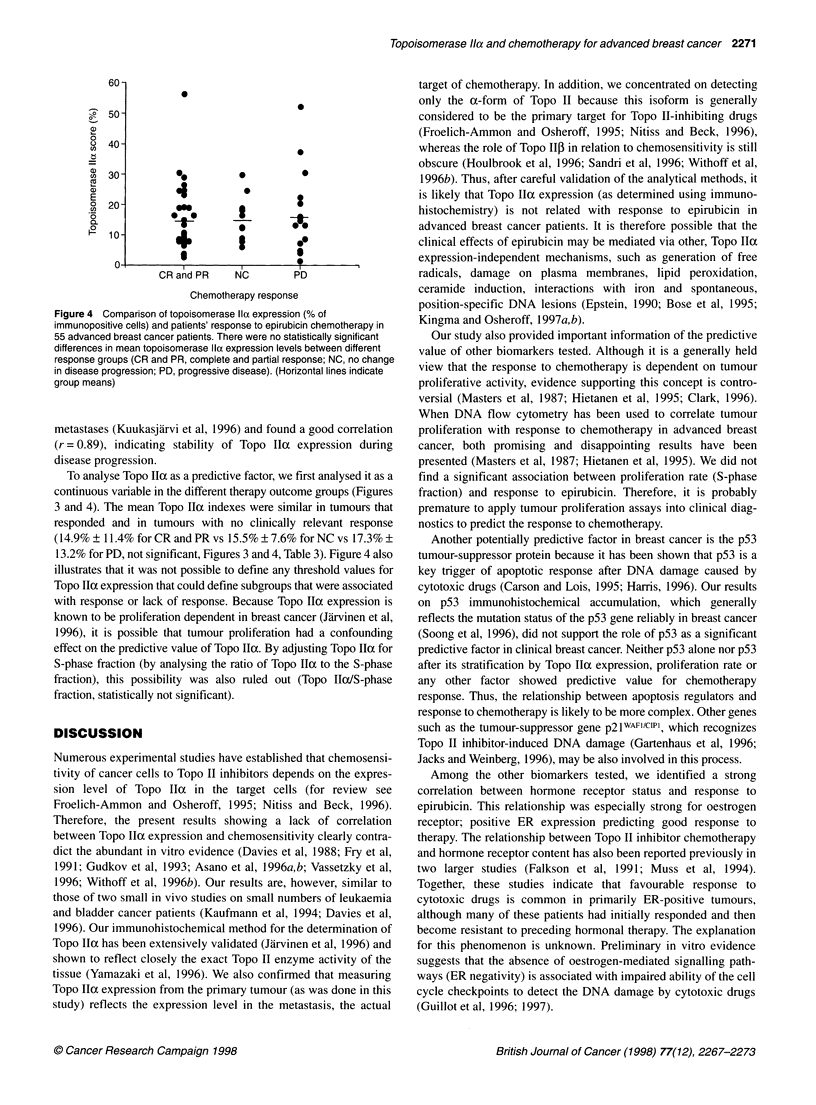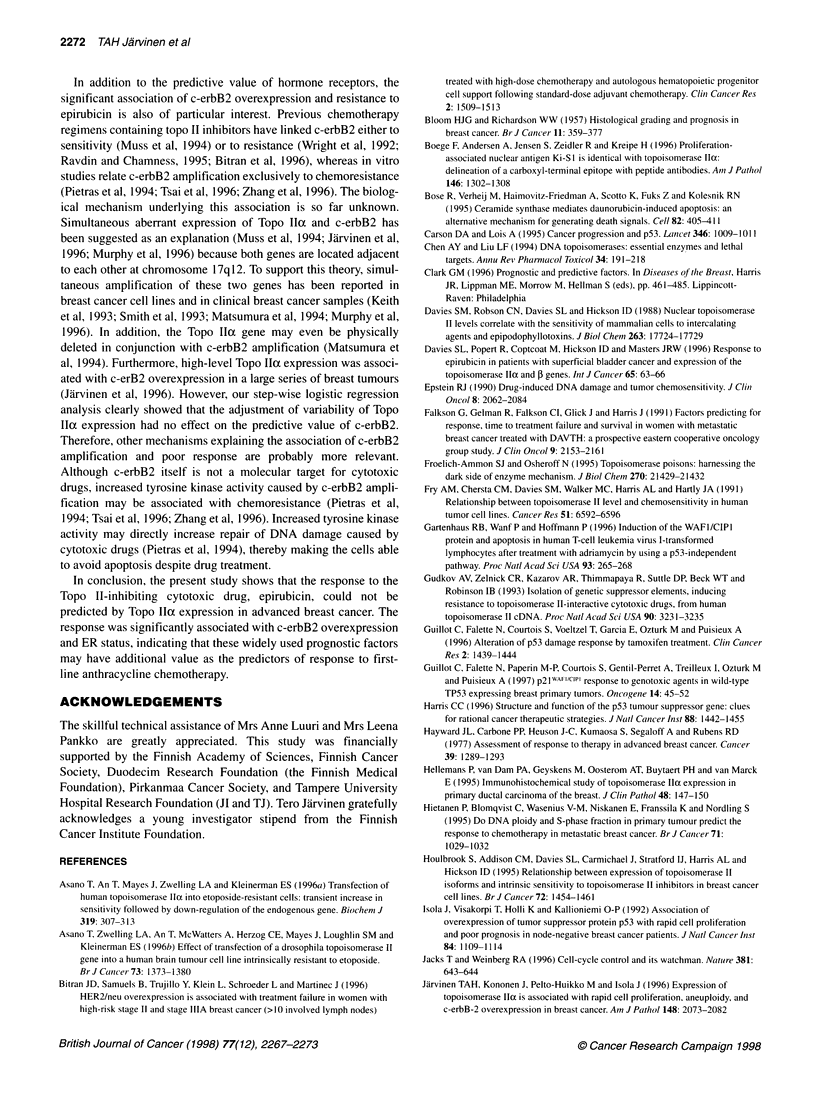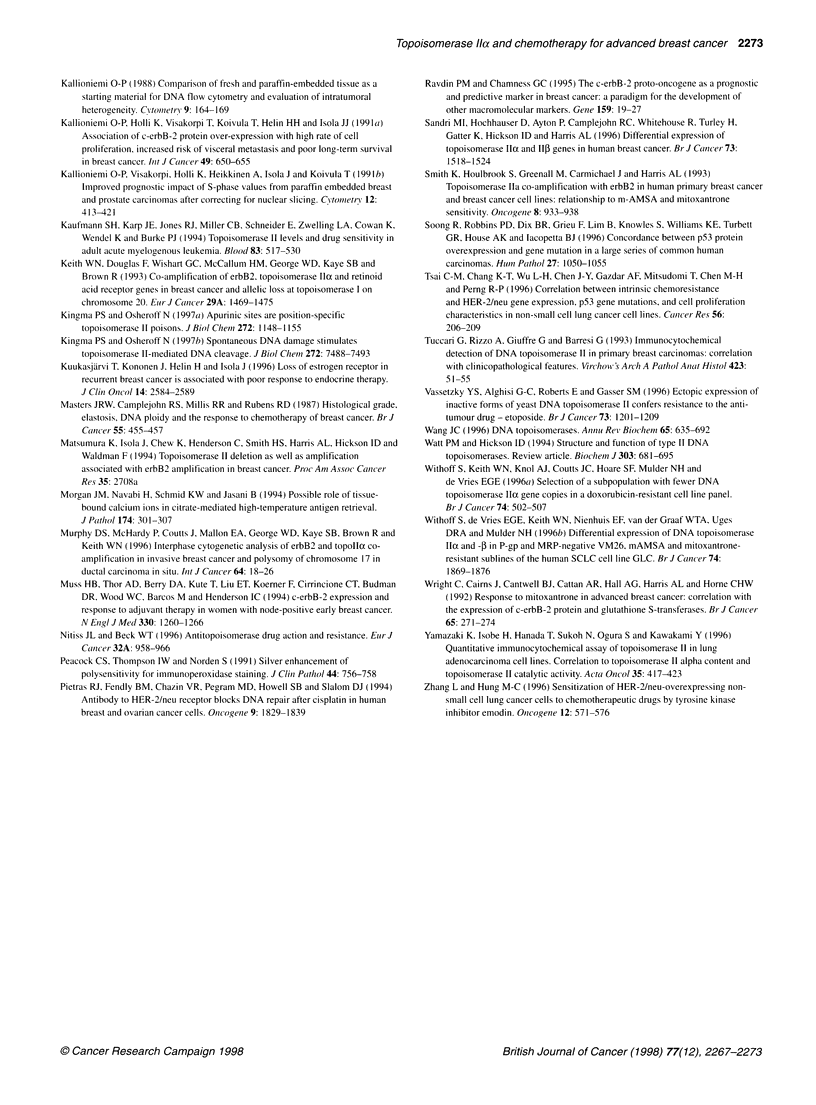# Predictive value of topoisomerase IIalpha and other prognostic factors for epirubicin chemotherapy in advanced breast cancer.

**DOI:** 10.1038/bjc.1998.377

**Published:** 1998-06

**Authors:** T. A. JÃ¤rvinen, K. Holli, T. KuukasjÃ¤rvi, J. J. Isola

**Affiliations:** Laboratory of Cancer Genetics, Tampere University Hospital and Institute of Medical Technology, University of Tampere, Finland.

## Abstract

**Images:**


British Journal of Cancer (1998) 77(12), 2267–2273
© 1998 Cancer Research Campaign

### Predictive value of topoisomerase IIα and other prognostic factors for epirubicin chemotherapy in advanced breast cancer

**TAH Järvinen[1], K Holli[2], T Kuukasjärvi[1,2] and JJ Isola[1]**

[1]Laboratory of Cancer Genetics, Tampere University Hospital and Institute of Medical Technology, University of Tampere, PO Box 607, FIN-33101 Tampere, Finland; [2]Departments of Oncology and Pathology, Tampere University Hospital, PO Box 2000, FIN-33521 Tampere, Finland

**Summary** Although cytotoxic chemotherapy is widely used in advanced breast cancer, there are no powerful predictors for the therapy response. Because topoisomerase IIα (Topo IIα) is the molecular target for the anthracycline class of anti-cancer drugs, we compared the immunocytochemical assay of Topo IIα with other biomarkers in the prediction of clinical response to Topo II inhibitor chemotherapy. Fifty-five patients with advanced breast cancer were treated with a single cytotoxic drug, Topo II-inhibitor, epirubicin (30 mg $m^{-2}$ weekly up to 1000 mg $m^{-2}$), as first line cytotoxic chemotherapy. Objective response to treatment was analysed according to UICC criteria. The predictive value of Topo IIα expression, c-erbB2 oncoprotein, p53 tumour-suppressor protein, oestrogen (ER) and progesterone receptor (PR), S-phase fraction and DNA ploidy were analysed from representative formalin-fixed paraffin-embedded primary tumour samples. The proportion of Topo IIα-positive cells (Topo IIα index) failed to predict response to epirubicin therapy. Mean Topo IIα scores in 29 responding patients were similar when compared with those with no change in disease progression ($n$ = 13) and those with progressive disease ($n$ = 13) (14.9% ± 11.4% vs 15.5% ± 7.6% vs 17.3% ± 13.2%, not significant). Among the other biomarkers tested, overexpression of c-erbB2 oncoprotein and hormone receptor negativity were significantly associated with poor response. Response rate in patients with c-erbB2-overexpressing tumours was 32% compared with 65% in patients with no c-erbB2 overexpression ($P$ = 0.0058). Accordingly, the response rate for ER-positive patients was 67% compared with 26% in ER-negative patients ($P$ = 0.0021). Although both negative ER status and c-erbB2 overexpression are associated with high Topo IIα expression in breast cancer, step-wise logistic regression analysis showed that ER and c-erbB2 were associated with therapy response independent of Topo IIα expression. Histological grade, p53, DNA-ploidy, tumour proliferation rate (S-phase fraction), stage of the disease at diagnosis, age of the patient, previous anti-oestrogen therapy or site of metastasis did not predict the response to epirubicin therapy. In conclusion, despite extensive in vitro evidence, expression of Topo IIα is unlikely to predict the response to Topo II inhibitor chemotherapy in advanced breast cancer. Among the prognostic biomarkers, overexpression of c-erbB2 oncogene and negative ER may have predictive value in epirubicin therapy in patients with advanced breast cancer.

**Keywords:** chemotherapy; breast cancer; topoisomerase IIα; c-erbB2 oncogene; DNA flow cytometry; hormone receptor

Patients with advanced breast cancer are commonly treated with cytotoxic chemotherapy. Whereas response to endocrine therapy can be effectively predicted by hormone receptor status, prediction of response to cytotoxic chemotherapy currently lacks reliable predictive markers (Clark, 1996). Such factors would be of utmost use, not only in the prediction of response to chemotherapy in general but also in aiding selection between different types of cytotoxic chemotherapy. With increasing number of effective chemotherapeutic agents, selection between different regimens for individual patients according to the predictive factors might improve the efficiency of chemotherapy.

Chemotherapy with anthracyclines (doxorubicin and its derivatives, such as epirubicin) is well established in advanced breast cancer. The mechanism of action of these compounds is related to the inhibition of topoisomerase II (Topo II) enzyme. Topo II is a eukaryotic homodimeric enzyme that exists in two isoforms in human cells: the 170-kDa form (Topo IIα) and the 180-kDa form (Topo IIβ). Whereas Topo IIα is a key enzyme in DNA metabolism, having key roles in DNA replication and chromosome partitioning during cell division, the function of Topo IIβ is poorly defined (Chen and Liu, 1994; Watt and Hickson, 1994; Froelich-Ammon and Osheroff, 1995; Wang, 1996). Drugs targeted against Topo II interfere with its DNA cleavage-rejoining action, trapping Topo IIα enzyme with DNA in a non-cleavable complex (Chen and Liu, 1994; Watt and Hickson, 1994; Froelich-Ammon and Osheroff, 1995). This results in the accumulation of stabilized double-stranded DNA breaks, which are lethal to the cell at the $G_2$M-phase of the cell cycle (Froelich-Ammon and Osheroff, 1995; Nitiss and Beck, 1996).

In vitro studies using different experimental designs have shown that sensitivity to Topo II-inhibiting drugs is dependent on the expression level of Topo IIα gene in target cancer cells (Davies et al, 1988; Fry et al, 1991; Gudkov et al, 1993; Asano et al, 1996*a*,*b*; Vassetzky et al, 1996; Withoff et al, 1996*a*,*b*). Cells with a low concentration of Topo IIα protein form fewer Topo II-mediated DNA strand breaks and are less sensitive to Topo II-inhibiting drugs than cells containing a high concentration of Topo IIα (Davies et al, 1988; Fry et al, 1991; Gudkov et al, 1993; Asano et al, 1996*a*,*b*; Vassetzky et al, 1996; Withoff et al, 1996*b*). These in vitro findings suggest testing whether assays of Topo IIα protein

*Received 30 April 1997*
*Revised 27 November 1997*
*Accepted 3 December 1997*

*Correspondence to:* T Järvinen expression could be used in a clinical setting to predict a patient's response to Topo II inhibitor chemotherapy. To date, no such clinical studies reporting the relationship between Topo IIα expression and chemosensitivity have been published using solid tumours, although studies on the variability of Topo IIα expression in breast tumours have been published (Tuccari et al, 1993; Hellemans et al, 1995; Boege et al, 1996; Järvinen et al, 1996; Sandri et al, 1996). In general, high Topo IIα expression is associated with high cellular proliferation and poor histological differentiation of the tumour (Tuccari et al, 1994; Hellemans et al, 1995; Boege et al, 1996; Järvinen et al, 1996; Sandri et al, 1996). In addition, our recent study on a large number of breast carcinomas indicated that Topo IIα expression also correlates with negative hormone receptor content, aneuploidy and c-erbB2 overexpression (Järvinen et al, 1996). These all are features that are generally considered to be related with altered sensitivity to chemotherapeutic agents.

In the present study, we compared the value of Topo IIα with other biomarkers (c-erbB2, p53, ER, PR, S-phase fraction, DNA ploidy) in the prediction of response to first-line Topo II inhibitor chemotherapy in 55 patients with advanced breast cancer.

**Figure 1** The survival of 55 advanced breast cancer patients after initiation of epirubicin according to the clinical response. Patients with complete or partial response (CR and PR) have significantly better survival than patients with no change in disease progression (NC) or progressive disease (PD) ($P < 0.0016$, Mantel–Cox test)

### MATERIALS AND METHODS

### Patients and tumours

Included in this study were 55 patients with advanced breast cancer treated with a single-agent chemotherapy programme comprising Topo II inhibitor, epirubicin (30 mg m$^{-2}$ weekly up to 1000 mg m$^{-2}$, for at least 3 months or until the disease progressed) between 1989 and 1995 in Tampere University Hospital. All patients received the therapy according to the schedule (no cessation of therapy due to side-effects). Epirubicin was the first-line cytotoxic chemotherapy for all patients, but 36 patients had received hormonal therapy (tamoxifen 20–40 mg day$^{-1}$) before epirubicin. Primary breast cancers of these patients were diagnosed and operated between 1981 and 1994, and the disease-free interval varied from 1 to 12.5 years. Twenty-five patients were primarily node negative (T1–3N0M0), 28 were node positive (T1–4N1M0), and in two patients the disease was advanced when diagnosis was made (T3N1M1). All patients were followed up every 3 weeks by clinical examination and laboratory tests (haemoglobin, platelets, alanine aminotransferase, alkaline phosphatase). Further diagnostic tests (bone scan, chest radiograph, liver ultrasound and serum tumour marker CA-15-3) were performed every 3 months to aid in determining the response to treatment. The response to chemotherapy was classified into four categories: 'complete response,' CR; 'partial response,' PR; 'no change in disease progression,' NC; and 'progressive disease,' PD according to UICC criteria (Hayward et al, 1977). The investigation was approved by the ethics committee of Tampere University Hospital and informed consent was obtained from each subject before enrolment to the study.

### Specimen preparation

Routinely formalin-fixed, paraffin-embedded blocks from the primary tumour lesions were obtained from the institutes in which the patients were operated. All histopathological diagnoses were re-evaluated and histopathological grading was performed according to the Bloom and Richardson system (Bloom and Richardson, 1957). Adjacent tissue sections were prepared for immunohistochemical studies, and 50-μm sections for flow cytometric DNA analysis.

### Immunohistochemistry

Immunohistochemistry for Topo IIα was performed using a rabbit polyclonal Topo IIα antibody (Topo IIα, diluted 1:1000 from manusfacturer's stock, TopoGEN, Columbus, OH, USA) (Järvinen et al, 1996). Tissue sections were cut on adhesive-treated, poly-L-lysine-coated slides and dried in an oven, 37°C, overnight and 3 h in an oven at 60°C. Dewaxed sections were immersed in 10 mM EDTA (pH 8.0) at 80°C and incubated at 120°C (pressure 1.05 bar) for 10 min in an ordinary autoclave for antigen retrieval (Morgan et al, 1994; Kuukasjärvi et al, 1996). Slides were washed at room temperature and incubated overnight at 4°C with the primary antibody. A standard avidin–biotin–peroxidase complex (ABC) technique (Vectastain Elite, Vector Laboratories, Burlingame, CA, USA) was used for visualization with diaminobenzidine as a chromogen. The diaminobenzidine reaction was intensified with the methenamine silver method as described elsewhere (Peacock et al, 1991). Sections were counterstained with haematoxylin and mounted. Immunostaining was evaluated light microscopically using an 20× objective, by a person unaware of clinical data. The Topo IIα index was assessed by counting the percentage of Topo IIα-positive cells from 400 to 1000 (average 600) carcinoma cells from a morphologically well-preserved area. The immunohistochemistry for c-erbB2, p53 and hormone receptors was performed on adjacent tissue sections as described previously (Kallioniemi et al, 1991*a*; Isola et al, 1992; Kuukasjärvi et al, 1996). Briefly, the immunohistochemistry of c-erbB2, p53 and hormone receptors was carried out essentially as described above for Topo IIα. Only intense membranous immunostaining present in a majority of cells was taken to represent overexpression of the c-erbB2 protein. ER, PR and p53-immunostaining in more than 20% of the tumour cells was regarded as immunopositivity (Kallioniemi et al, 1991; Isola et al, 1992; Kuukasjärvi et al, 1996).

© Cancer Research Campaign 1998

**Table 1** Association of clinical response to epirubicin-chemotherapy with clinicopathological variables in 55 advanced breast cancer patients

| Variable | CR and PR | NC | PD | *P*-value[a] |
|---|---|---|---|---|
| All tumours | 53% (29/55) | 18% (10/55) | 29% (16/55) | |
| Age | | | | |
| < 50 years | 51% (18/35) | 17% (6/35) | 31% (11/35) | |
| > 50 years | 55% (11/20) | 20% (4/20) | 25% (5/20) | NS |
| Histological grade | | | | |
| I or II | 43% (13/30) | 27% (8/30) | 30% (9/30) | |
| III | 64% (16/25) | 8% (2/25) | 28% (7/25) | NS |
| Oestrogen receptor[b] | | | | |
| Negative | 26% (5/19) | 21% (4/19) | 53% (10/19) | |
| Positive | 67% (24/36) | 17% (6/36) | 16% (6/36) | 0.0021 |
| Progesterone receptor[b] | | | | |
| Negative | 43% (12/28) | 14% (4/28) | 43% (12/28) | |
| Positive | 63% (17/27) | 22% (6/27) | 15% (4/27) | 0.0409 |
| Topoisomerase IIα | | | | |
| < 15% | 58% (18/31) | 16% (5/31) | 26% (8/31) | |
| > 15% | 46% (11/24) | 21% (5/24) | 33% (8/24) | NS |
| C-erbB-2 overexpression | | | | |
| Negative | 64% (23/36) | 19% (7/36) | 17% (6/36) | |
| Positive | 32% (6/19) | 16% (3/19) | 53% (10/19) | 0.0058 |
| p53 overexpression | | | | |
| Negative | 57% (21/37) | 19% (7/37) | 24% (9/37) | |
| Mutated | 44% (8/18) | 17% (3/18) | 39% (7/18) | NS |
| DNA ploidy | | | | |
| Diploid | 55% (12/22) | 22% (5/22) | 22% (5/22) | |
| Aneuploid | 50% (16/32) | 16% (5/32) | 34% (11/32) | NS |
| S-phase fraction | | | | |
| < 8% | 55% (11/20) | 25% (5/20) | 20% (4/20) | |
| > 8% | 50% (16/32) | 25% (4/16) | 25% (4/16) | NS |
| Site of metastasis | | | | |
| Bone | 50% (11/22) | 9% (2/22) | 41% (10/22) | |
| Lung | 0% (0/3) | 100% (3/3) | 0% (0/3) | |
| Liver | 50% (4/8) | 0% (0/8) | 50% (4/8) | |
| All other sites | 80% (4/5) | 20% (1/5) | 0% (0/5) | |
| Multiple sites | 63% (10/16) | 25% (4/16) | 22% (2/16) | NS |

[a]*P*-value for chi-square test for linear trend. [b]Negative, < 20% immunopositive cells; positive, ≥ 20% immunopositive cells. NS, not significant.

**Figure 2** Correlation of the immunohistochemical determination of topoisomerase IIα protein expression between frozen and formalin-fixed, paraffin-embedded tissue sections. A highly significant correlation was found ($r = 0.89$) in 20 tumours, validating the use of paraffin sections as study material

Our immunohistochemical determination of Topo IIα on frozen sections has been previously validated using Western blotting, dual-colour immunofluorescence and mRNA in situ hybridization (Järvinen et al, 1996). Now, the applicability of this assay was extended to formalin-fixed paraffin-embedded tissue sections by comparing Topo IIα expression from the randomly selected, separate set of 20 breast tumours from which both frozen and formalin-fixed, paraffin-embedded tissue sections were available.

Because only primary tumours were available from epirubicin-treated patients, we studied the correlation of Topo IIα expression in the primary and metastatic tumours in a separate set of eight pairs of primary and metastatic breast carcinomas (Kuukasjärvi et al, 1996). Immunohistochemical controls for the ER, PR, p53 and c-erbB2 immunostainings have been established previously (Kallioniemi et al, 1991*a*; Isola et al, 1992; Kuukasjärvi et al, 1996).

### DNA flow cytometry

DNA flow cytometry was performed using dewaxed, rehydrated and trypsin-treated, 50-μm-thick, paraffin-embedded sections as the starting material, using protocols described previously (Kallioniemi, 1988). Flow cytometric analysis was carried out using an EPICS C flow cytometer (Coulter Electronics, Hialeh, FL, USA) and the MultiCycle software for data analysis (Phoenix Flow Systems, San Diego, CA, USA). In DNA aneuploid histograms, the S-phase was analysed only from the aneuploid clone. A sliced nuclei background subtraction was performed in all cases to compensate for the effects of nuclear debris on cell cycle distribution (Kallioniemi et al, 1991*b*).

### Statistical methods

Statistical analyses were carried out with an IBM-compatible personal computer and the Biomedical Data Processing Software (BMDP Statistical Software, Los Angeles, CA, USA). The survival analysis was performed using the Mantel–Cox test (program 1L). The association between chemosensitivity with prognostic factors was performed using 2 × 3 frequency tables (using chi-square test for linear trend) (program 4F). The independent predictive value of each marker was tested using step-wise logistic regression analysis (program LR). The association of chemosensitivity with Topo IIα score and S-phase fraction was also performed using one-way analysis of variance (ANOVA with the Welch correction for unequal variances) (program 7D).

### RESULTS

Among the 55 patients treated with epirubicin, there were two CRs (4%), 27 PRs (49%), ten patients with NC (18%) and 16 patients with PD (29%). For all further analyses the two complete responders were combined together with the patients with partial response (CR and PR, $n = 29$). The follow-up of patients showed that survival after administration of epirubicin was significantly better in the favourably responding group (CR and PR) than in patients with NC or with PD ($P < 0.0016$, Figure 1). Treatment

© Cancer Research Campaign 1998

**Table 2** Step-wise logistic regression analysis of independent predictive factors of response to epirubicin chemotherapy in 55 patients with advanced breast cancer

| Variable | Relative risk of no response (95% confidence interval) | *P*-value |
|---|---|---|
| Oestrogen receptor[a] (negative vs positive) | 8.7 (1.4–53) | 0.012 |
| C-erbB2 overexpression (negative vs positive) | 5.4 (0.8–35) | 0.063 |

Co-variates included topo IIα, p53, PR, S-phase fraction, grade, DNA ploidy.
[a]Negative, < 20% immunopositive cells; positive, ≥20% immunopositive cells.

**Table 3** Association of clinical response to epirubicin chemotherapy with topoisomerase IIα score and S-phase fraction in 55 advanced breast cancer patients

| | Response to therapy | | | |
|---|---|---|---|---|
| Variable | CR and PR ($n = 29$) | NC ($n = 10$) | PD ($n = 16$) | *P*-value[a] |
| Topoisomerase IIα | 14.9 ± 11.4 | 15.5 ± 7.6 | 17.3 ± 13.2 | NS |
| S-phase fraction | 11.0 ± 7.2 | 11.6 ± 9.2 | 11.5 ± 6.3 | NS |

[a]ANOVA. NS, not significant.

before epirubicin therapy did not affect the results because epirubicin was used as the first-line chemotherapy (no prior cytotoxic drugs). Previous anti-oestrogen therapy (in 36 patients) was not associated with response to epirubicin ($P = 0.20$).

The association between response to epirubicin and various clinicopathological factors was examined as shown in Table 1. None of the clinicopathological variables (age of patient, size of primary tumour, axillary lymph node status, site of metastasis in advanced disease and histological grade) was associated with response ($P > 0.05$ for all variables). Among the biomarkers studied, c-erbB2 oncoprotein overexpression ($P = 0.0058$), negative ER ($P = 0.0021$) and PR status ($P = 0.041$) were statistically significantly associated with the lack of response to chemotherapy (Table 1). p53 tumour-suppressor protein accumulation, DNA ploidy or tumour proliferation rate (S-phase fraction) were not associated with clinical response to chemotherapy (Table 1). A step-wise logistic regression analysis was used to demonstrate that the predictive value of ER and c-erB2 were independent of the Topo IIα score and other clinical and biological variables studied (Table 2). The tumour proliferation rate (S-phase fraction) was also analysed as a continuous variable (Table 3). Mean S-phase fractions in responders and non-responders were similar (11.0% ± 7.2% for CR and PR vs 11.6% ± 9.2% for NC vs 11.5% ± 6.3% for PD, not significant, Table 3).

The relationship between response to epirubicin and Topo IIα expression was the main interest in this study and was studied in detail. First, we validated our immunohistochemical Topo IIα assay for formalin-fixed, paraffin-embedded sections by comparing them with those from adjacent frozen sections used previously (Järvinen et al, 1996). Topo IIα scores that showed excellent correlation were found between these two sample types (correlation coefficient, $r = 0.89$, $n = 20$, Figure 2). Next, we confirmed that Topo IIα expression measured in a primary tumour sample reflects the Topo IIα status of metastasis, which is the target of chemotherapy but from which biopsies were not available. We analysed eight pairs of primary tumour and asynchronous

**Figure 3** Lack of correlation between immunohistochemically determined topoisomerase IIα expression and response to epirubicin in four human breast carcinomas. Both high and low topoisomerase IIα scores were found in tumours from the patients with favourable response (A and C, topoisomerase IIα scores 56.2% and 3.7% respectively) or from those with progressive disease despite epirubicin therapy (B and D, topoisomerase IIα scores 52.1% and 3.8%). Haematoxylin counterstain; magnification × 240

© Cancer Research Campaign 1998

**Figure 4** Comparison of topoisomerase IIα expression (% of immunopositive cells) and patients' response to epirubicin chemotherapy in 55 advanced breast cancer patients. There were no statistically significant differences in mean topoisomerase IIα expression levels between different response groups (CR and PR, complete and partial response; NC, no change in disease progression; PD, progressive disease). (Horizontal lines indicate group means)

metastases (Kuukasjärvi et al, 1996) and found a good correlation ($r = 0.89$), indicating stability of Topo IIα expression during disease progression.

To analyse Topo IIα as a predictive factor, we first analysed it as a continuous variable in the different therapy outcome groups (Figures 3 and 4). The mean Topo IIα indexes were similar in tumours that responded and in tumours with no clinically relevant response (14.9% ± 11.4% for CR and PR vs 15.5% ± 7.6% for NC vs 17.3% ± 13.2% for PD, not significant, Figures 3 and 4, Table 3). Figure 4 also illustrates that it was not possible to define any threshold values for Topo IIα expression that could define subgroups that were associated with response or lack of response. Because Topo IIα expression is known to be proliferation dependent in breast cancer (Järvinen et al, 1996), it is possible that tumour proliferation had a confounding effect on the predictive value of Topo IIα. By adjusting Topo IIα for S-phase fraction (by analysing the ratio of Topo IIα to the S-phase fraction), this possibility was also ruled out (Topo IIα/S-phase fraction, statistically not significant).

### DISCUSSION

Numerous experimental studies have established that chemosensitivity of cancer cells to Topo II inhibitors depends on the expression level of Topo IIα in the target cells (for review see Froelich-Ammon and Osheroff, 1995; Nitiss and Beck, 1996). Therefore, the present results showing a lack of correlation between Topo IIα expression and chemosensitivity clearly contradict the abundant in vitro evidence (Davies et al, 1988; Fry et al, 1991; Gudkov et al, 1993; Asano et al, 1996*a,b*; Vassetzky et al, 1996; Withoff et al, 1996*b*). Our results are, however, similar to those of two small in vivo studies on small numbers of leukaemia and bladder cancer patients (Kaufmann et al, 1994; Davies et al, 1996). Our immunohistochemical method for the determination of Topo IIα has been extensively validated (Järvinen et al, 1996) and shown to reflect closely the exact Topo II enzyme activity of the tissue (Yamazaki et al, 1996). We also confirmed that measuring Topo IIα expression from the primary tumour (as was done in this study) reflects the expression level in the metastasis, the actual target of chemotherapy. In addition, we concentrated on detecting only the α-form of Topo II because this isoform is generally considered to be the primary target for Topo II-inhibiting drugs (Froelich-Ammon and Osheroff, 1995; Nitiss and Beck, 1996), whereas the role of Topo IIβ in relation to chemosensitivity is still obscure (Houlbrook et al, 1996; Sandri et al, 1996; Withoff et al, 1996*b*). Thus, after careful validation of the analytical methods, it is likely that Topo IIα expression (as determined using immunohistochemistry) is not related with response to epirubicin in advanced breast cancer patients. It is therefore possible that the clinical effects of epirubicin may be mediated via other, Topo IIα expression-independent mechanisms, such as generation of free radicals, damage on plasma membranes, lipid peroxidation, ceramide induction, interactions with iron and spontaneous, position-specific DNA lesions (Epstein, 1990; Bose et al, 1995; Kingma and Osheroff, 1997*a,b*).

Our study also provided important information of the predictive value of other biomarkers tested. Although it is a generally held view that the response to chemotherapy is dependent on tumour proliferative activity, evidence supporting this concept is controversial (Masters et al, 1987; Hietanen et al, 1995; Clark, 1996). When DNA flow cytometry has been used to correlate tumour proliferation with response to chemotherapy in advanced breast cancer, both promising and disappointing results have been presented (Masters et al, 1987; Hietanen et al, 1995). We did not find a significant association between proliferation rate (S-phase fraction) and response to epirubicin. Therefore, it is probably premature to apply tumour proliferation assays into clinical diagnostics to predict the response to chemotherapy.

Another potentially predictive factor in breast cancer is the p53 tumour-suppressor protein because it has been shown that p53 is a key trigger of apoptotic response after DNA damage caused by cytotoxic drugs (Carson and Lois, 1995; Harris, 1996). Our results on p53 immunohistochemical accumulation, which generally reflects the mutation status of the p53 gene reliably in breast cancer (Soong et al, 1996), did not support the role of p53 as a significant predictive factor in clinical breast cancer. Neither p53 alone nor p53 after its stratification by Topo IIα expression, proliferation rate or any other factor showed predictive value for chemotherapy response. Thus, the relationship between apoptosis regulators and response to chemotherapy is likely to be more complex. Other genes such as the tumour-suppressor gene $p21^{WAF1/CIP1}$, which recognizes Topo II inhibitor-induced DNA damage (Gartenhaus et al, 1996; Jacks and Weinberg, 1996), may be also involved in this process.

Among the other biomarkers tested, we identified a strong correlation between hormone receptor status and response to epirubicin. This relationship was especially strong for oestrogen receptor; positive ER expression predicting good response to therapy. The relationship between Topo II inhibitor chemotherapy and hormone receptor content has also been reported previously in two larger studies (Falkson et al, 1991; Muss et al, 1994). Together, these studies indicate that favourable response to cytotoxic drugs is common in primarily ER-positive tumours, although many of these patients had initially responded and then become resistant to preceding hormonal therapy. The explanation for this phenomenon is unknown. Preliminary in vitro evidence suggests that the absence of oestrogen-mediated signalling pathways (ER negativity) is associated with impaired ability of the cell cycle checkpoints to detect the DNA damage by cytotoxic drugs (Guillot et al, 1996; 1997).

In addition to the predictive value of hormone receptors, the significant association of c-erbB2 overexpression and resistance to epirubicin is also of particular interest. Previous chemotherapy regimens containing topo II inhibitors have linked c-erbB2 either to sensitivity (Muss et al, 1994) or to resistance (Wright et al, 1992; Ravdin and Chamness, 1995; Bitran et al, 1996), whereas in vitro studies relate c-erbB2 amplification exclusively to chemoresistance (Pietras et al, 1994; Tsai et al, 1996; Zhang et al, 1996). The biological mechanism underlying this association is so far unknown. Simultaneous aberrant expression of Topo IIα and c-erbB2 has been suggested as an explanation (Muss et al, 1994; Järvinen et al, 1996; Murphy et al, 1996) because both genes are located adjacent to each other at chromosome 17q12. To support this theory, simultaneous amplification of these two genes has been reported in breast cancer cell lines and in clinical breast cancer samples (Keith et al, 1993; Smith et al, 1993; Matsumura et al, 1994; Murphy et al, 1996). In addition, the Topo IIα gene may even be physically deleted in conjunction with c-erbB2 amplification (Matsumura et al, 1994). Furthermore, high-level Topo IIα expression was associated with c-erB2 overexpression in a large series of breast tumours (Järvinen et al, 1996). However, our step-wise logistic regression analysis clearly showed that the adjustment of variability of Topo IIα expression had no effect on the predictive value of c-erbB2. Therefore, other mechanisms explaining the association of c-erbB2 amplification and poor response are probably more relevant. Although c-erbB2 itself is not a molecular target for cytotoxic drugs, increased tyrosine kinase activity caused by c-erbB2 amplification may be associated with chemoresistance (Pietras et al, 1994; Tsai et al, 1996; Zhang et al, 1996). Increased tyrosine kinase activity may directly increase repair of DNA damage caused by cytotoxic drugs (Pietras et al, 1994), thereby making the cells able to avoid apoptosis despite drug treatment.

In conclusion, the present study shows that the response to the Topo II-inhibiting cytotoxic drug, epirubicin, could not be predicted by Topo IIα expression in advanced breast cancer. The response was significantly associated with c-erbB2 overexpression and ER status, indicating that these widely used prognostic factors may have additional value as the predictors of response to first-line anthracycline chemotherapy.

### ACKNOWLEDGEMENTS

The skillful technical assistance of Mrs Anne Luuri and Mrs Leena Pankko are greatly appreciated. This study was financially supported by the Finnish Academy of Sciences, Finnish Cancer Society, Duodecim Research Foundation (the Finnish Medical Foundation), Pirkanmaa Cancer Society, and Tampere University Hospital Research Foundation (JI and TJ). Tero Järvinen gratefully acknowledges a young investigator stipend from the Finnish Cancer Institute Foundation.

### REFERENCES

Asano T, An T, Mayes J, Zwelling LA and Kleinerman ES (1996*a*) Transfection of human topoisomerase IIα into etoposide-resistant cells: transient increase in sensitivity followed by down-regulation of the endogenous gene. *Biochem J* **319**: 307–313

Asano T, Zwelling LA, An T, McWatters A, Herzog CE, Mayes J, Loughlin SM and Kleinerman ES (1996*b*) Effect of transfection of a drosophila topoisomerase II gene into a human brain tumour cell line intrinsically resistant to etoposide. *Br J Cancer* **73**: 1373–1380

Bitran JD, Samuels B, Trujillo Y, Klein L, Schroeder L and Martinec J (1996) HER2/neu overexpression is associated with treatment failure in women with high-risk stage II and stage IIIA breast cancer (>10 involved lymph nodes) treated with high-dose chemotherapy and autologous hematopoietic progenitor cell support following standard-dose adjuvant chemotherapy. *Clin Cancer Res* **2**: 1509–1513

Bloom HJG and Richardson WW (1957) Histological grading and prognosis in breast cancer. *Br J Cancer* **11**: 359–377

Boege F, Andersen A, Jensen S, Zeidler R and Kreipe H (1996) Proliferation-associated nuclear antigen Ki-S1 is identical with topoisomerase IIα: delineation of a carboxyl-terminal epitope with peptide antibodies. *Am J Pathol* **146**: 1302–1308

Bose R, Verheij M, Haimovitz-Friedman A, Scotto K, Fuks Z and Kolesnik RN (1995) Ceramide synthase mediates daunorubicin-induced apoptosis: an alternative mechanism for generating death signals. *Cell* **82**: 405–411

Carson DA and Lois A (1995) Cancer progression and p53. *Lancet* **346**: 1009–1011

Chen AY and Liu LF (1994) DNA topoisomerases: essential enzymes and lethal targets. *Annu Rev Pharmacol Toxicol* **34**: 191–218

Clark GM (1996) Prognostic and predictive factors. In *Diseases of the Breast*, Harris JR, Lippman ME, Morrow M, Hellman S (eds), pp. 461–485. Lippincott-Raven: Philadelphia

Davies SM, Robson CN, Davies SL and Hickson ID (1988) Nuclear topoisomerase II levels correlate with the sensitivity of mammalian cells to intercalating agents and epipodophyllotoxins. *J Biol Chem* **263**: 17724–17729

Davies SL, Popert R, Coptcoat M, Hickson ID and Masters JRW (1996) Response to epirubicin in patients with superficial bladder cancer and expression of the topoisomerase IIα and β genes. *Int J Cancer* **65**: 63–66

Epstein RJ (1990) Drug-induced DNA damage and tumor chemosensitivity. *J Clin Oncol* **8**: 2062–2084

Falkson G, Gelman R, Falkson CI, Glick J and Harris J (1991) Factors predicting for response, time to treatment failure and survival in women with metastatic breast cancer treated with DAVTH: a prospective eastern cooperative oncology group study. *J Clin Oncol* **9**: 2153–2161

Froelich-Ammon SJ and Osheroff N (1995) Topoisomerase poisons: harnessing the dark side of enzyme mechanism. *J Biol Chem* **270**: 21429–21432

Fry AM, Chersta CM, Davies SM, Walker MC, Harris AL and Hartly JA (1991) Relationship between topoisomerase II level and chemosensitivity in human tumor cell lines. *Cancer Res* **51**: 6592–6596

Gartenhaus RB, Wanf P and Hoffmann P (1996) Induction of the WAF1/CIP1 protein and apoptosis in human T-cell leukemia virus I-transformed lymphocytes after treatment with adriamycin by using a p53-independent pathway. *Proc Natl Acad Sci USA* **93**: 265–268

Gudkov AV, Zelnick CR, Kazarov AR, Thimmapaya R, Suttle DP, Beck WT and Robinson IB (1993) Isolation of genetic suppressor elements, inducing resistance to topoisomerase II-interactive cytotoxic drugs, from human topoisomerase II cDNA. *Proc Natl Acad Sci USA* **90**: 3231–3235

Guillot C, Falette N, Courtois S, Voeltzel T, Garcia E, Ozturk M and Puisieux A (1996) Alteration of p53 damage response by tamoxifen treatment. *Clin Cancer Res* **2**: 1439–1444

Guillot C, Falette N, Paperin M-P, Courtois S, Gentil-Perret A, Treilleux I, Ozturk M and Puisieux A (1997) p21$^{WAF1/CIP1}$ response to genotoxic agents in wild-type TP53 expressing breast primary tumors. *Oncogene* **14**: 45–52

Harris CC (1996) Structure and function of the p53 tumour suppressor gene: clues for rational cancer therapeutic strategies. *J Natl Cancer Inst* **88**: 1442–1455

Hayward JL, Carbone PP, Heuson J-C, Kumaosa S, Segaloff A and Rubens RD (1977) Assessment of response to therapy in advanced breast cancer. *Cancer* **39**: 1289–1293

Hellemans P, van Dam PA, Geyskens M, Oosterom AT, Buytaert PH and van Marck E (1995) Immunohistochemical study of topoisomerase IIα expression in primary ductal carcinoma of the breast. *J Clin Pathol* **48**: 147–150

Hietanen P, Blomqvist C, Wasenius V-M, Niskanen E, Franssila K and Nordling S (1995) Do DNA ploidy and S-phase fraction in primary tumour predict the response to chemotherapy in metastatic breast cancer. *Br J Cancer* **71**: 1029–1032

Houlbrook S, Addison CM, Davies SL, Carmichael J, Stratford IJ, Harris AL and Hickson ID (1995) Relationship between expression of topoisomerase II isoforms and intrinsic sensitivity to topoisomerase II inhibitors in breast cancer cell lines. *Br J Cancer* **72**: 1454–1461

Isola J, Visakorpi T, Holli K and Kallioniemi O-P (1992) Association of overexpression of tumor suppressor protein p53 with rapid cell proliferation and poor prognosis in node-negative breast cancer patients. *J Natl Cancer Inst* **84**: 1109–1114

Jacks T and Weinberg RA (1996) Cell-cycle control and its watchman. *Nature* **381**: 643–644

Järvinen TAH, Kononen J, Pelto-Huikko M and Isola J (1996) Expression of topoisomerase IIα is associated with rapid cell proliferation, aneuploidy, and c-erbB-2 overexpression in breast cancer. *Am J Pathol* **148**: 2073–2082

© Cancer Research Campaign 1998

Kallioniemi O-P (1988) Comparison of fresh and paraffin-embedded tissue as a starting material for DNA flow cytometry and evaluation of intratumoral heterogeneity. *Cytometry* **9**: 164–169

Kallioniemi O-P, Holli K, Visakorpi T, Koivula T, Helin HH and Isola JJ (1991*a*) Association of c-erbB-2 protein over-expression with high rate of cell proliferation, increased risk of visceral metastasis and poor long-term survival in breast cancer. *Int J Cancer* **49**: 650–655

Kallioniemi O-P, Visakorpi, Holli K, Heikkinen A, Isola J and Koivula T (1991*b*) Improved prognostic impact of S-phase values from paraffin embedded breast and prostate carcinomas after correcting for nuclear slicing. *Cytometry* **12**: 413–421

Kaufmann SH, Karp JE, Jones RJ, Miller CB, Schneider E, Zwelling LA, Cowan K, Wendel K and Burke PJ (1994) Topoisomerase II levels and drug sensitivity in adult acute myelogenous leukemia. *Blood* **83**: 517–530

Keith WN, Douglas F, Wishart GC, McCallum HM, George WD, Kaye SB and Brown R (1993) Co-amplification of erbB2, topoisomerase IIα and retinoid acid receptor genes in breast cancer and allelic loss at topoisomerase I on chromosome 20. *Eur J Cancer* **29A**: 1469–1475

Kingma PS and Osheroff N (1997*a*) Apurinic sites are position-specific topoisomerase II poisons. *J Biol Chem* **272**: 1148–1155

Kingma PS and Osheroff N (1997*b*) Spontaneous DNA damage stimulates topoisomerase II-mediated DNA cleavage. *J Biol Chem* **272**: 7488–7493

Kuukasjärvi T, Kononen J, Helin H and Isola J (1996) Loss of estrogen receptor in recurrent breast cancer is associated with poor response to endocrine therapy. *J Clin Oncol* **14**: 2584–2589

Masters JRW, Camplejohn RS, Millis RR and Rubens RD (1987) Histological grade, elastosis, DNA ploidy and the response to chemotherapy of breast cancer. *Br J Cancer* **55**: 455–457

Matsumura K, Isola J, Chew K, Henderson C, Smith HS, Harris AL, Hickson ID and Waldman F (1994) Topoisomerase II deletion as well as amplification associated with erbB2 amplification in breast cancer. *Proc Am Assoc Cancer Res* **35**: 2708a

Morgan JM, Navabi H, Schmid KW and Jasani B (1994) Possible role of tissue-bound calcium ions in citrate-mediated high-temperature antigen retrieval. *J Pathol* **174**: 301–307

Murphy DS, McHardy P, Coutts J, Mallon EA, George WD, Kaye SB, Brown R and Keith WN (1996) Interphase cytogenetic analysis of erbB2 and topoIIα co-amplification in invasive breast cancer and polysomy of chromosome 17 in ductal carcinoma in situ. *Int J Cancer* **64**: 18–26

Muss HB, Thor AD, Berry DA, Kute T, Liu ET, Koerner F, Cirrincione CT, Budman DR, Wood WC, Barcos M and Henderson IC (1994) c-erbB-2 expression and response to adjuvant therapy in women with node-positive early breast cancer. *N Engl J Med* **330**: 1260–1266

Nitiss JL and Beck WT (1996) Antitopoisomerase drug action and resistance. *Eur J Cancer* **32A**: 958–966

Peacock CS, Thompson IW and Norden S (1991) Silver enhancement of polysensitivity for immunoperoxidase staining. *J Clin Pathol* **44**: 756–758

Pietras RJ, Fendly BM, Chazin VR, Pegram MD, Howell SB and Slalom DJ (1994) Antibody to HER-2/neu receptor blocks DNA repair after cisplatin in human breast and ovarian cancer cells. *Oncogene* **9**: 1829–1839

Ravdin PM and Chamness GC (1995) The c-erbB-2 proto-oncogene as a prognostic and predictive marker in breast cancer: a paradigm for the development of other macromolecular markers. *Gene* **159**: 19–27

Sandri MI, Hochhauser D, Ayton P, Camplejohn RC, Whitehouse R, Turley H, Gatter K, Hickson ID and Harris AL (1996) Differential expression of topoisomerase IIα and IIβ genes in human breast cancer. *Br J Cancer* **73**: 1518–1524

Smith K, Houlbrook S, Greenall M, Carmichael J and Harris AL (1993) Topoisomerase IIa co-amplification with erbB2 in human primary breast cancer and breast cancer cell lines: relationship to m-AMSA and mitoxantrone sensitivity. *Oncogene* **8**: 933–938

Soong R, Robbins PD, Dix BR, Grieu F, Lim B, Knowles S, Williams KE, Turbett GR, House AK and Iacopetta BJ (1996) Concordance between p53 protein overexpression and gene mutation in a large series of common human carcinomas. *Hum Pathol* **27**: 1050–1055

Tsai C-M, Chang K-T, Wu L-H, Chen J-Y, Gazdar AF, Mitsudomi T, Chen M-H and Perng R-P (1996) Correlation between intrinsic chemoresistance and HER-2/neu gene expression, p53 gene mutations, and cell proliferation characteristics in non-small cell lung cancer cell lines. *Cancer Res* **56**: 206–209

Tuccari G, Rizzo A, Giuffre G and Barresi G (1993) Immunocytochemical detection of DNA topoisomerase II in primary breast carcinomas: correlation with clinicopathological features. *Virchow's Arch A Pathol Anat Histol* **423**: 51–55

Vassetzky YS, Alghisi G-C, Roberts E and Gasser SM (1996) Ectopic expression of inactive forms of yeast DNA topoisomerase II confers resistance to the anti-tumour drug – etoposide. *Br J Cancer* **73**: 1201–1209

Wang JC (1996) DNA topoisomerases. *Annu Rev Biochem* **65**: 635–692

Watt PM and Hickson ID (1994) Structure and function of type II DNA topoisomerases. Review article. *Biochem J* **303**: 681–695

Withoff S, Keith WN, Knol AJ, Coutts JC, Hoare SF, Mulder NH and de Vries EGE (1996*a*) Selection of a subpopulation with fewer DNA topoisomerase IIα gene copies in a doxorubicin-resistant cell line panel. *Br J Cancer* **74**: 502–507

Withoff S, de Vries EGE, Keith WN, Nienhuis EF, van der Graaf WTA, Uges DRA and Mulder NH (1996*b*) Differential expression of DNA topoisomerase IIα and -β in P-gp and MRP-negative VM26, mAMSA and mitoxantrone-resistant sublines of the human SCLC cell line GLC. *Br J Cancer* **74**: 1869–1876

Wright C, Cairns J, Cantwell BJ, Cattan AR, Hall AG, Harris AL and Horne CHW (1992) Response to mitoxantrone in advanced breast cancer: correlation with the expression of c-erbB-2 protein and glutathione S-transferases. *Br J Cancer* **65**: 271–274

Yamazaki K, Isobe H, Hanada T, Sukoh N, Ogura S and Kawakami Y (1996) Quantitative immunocytochemical assay of topoisomerase II in lung adenocarcinoma cell lines. Correlation to topoisomerase II alpha content and topoisomerase II catalytic activity. *Acta Oncol* **35**: 417–423

Zhang L and Hung M-C (1996) Sensitization of HER-2/neu-overexpressing non-small cell lung cancer cells to chemotherapeutic drugs by tyrosine kinase inhibitor emodin. *Oncogene* **12**: 571–576

© Cancer Research Campaign 1998